# Identifying people with a learning disability: an advanced search for general practice

**DOI:** 10.3399/bjgp17X693461

**Published:** 2017-10-24

**Authors:** Amy M Russell, Louise Bryant, Allan House

**Affiliations:** Leeds Institute of Health Sciences, University of Leeds, Leeds.; Leeds Institute of Health Sciences, University of Leeds, Leeds.; Leeds Institute of Health Sciences, University of Leeds, Leeds.

**Keywords:** general practice, health checks, learning disorders, patient selection, primary health care, Read Code

## Abstract

**Background:**

People with learning disabilities (LD) have poor physical and mental health when compared with the general population. They are also likely to find it more difficult than others to describe their symptoms adequately. It is therefore harder for healthcare workers to identify the health needs of those with learning disabilities, with the danger of some problems being left unrecognised. Practice registers record only a proportion of those who are eligible, making it difficult to target improvements in their health care.

**Aim:**

To test a Read Code search supporting the identification of people with a mild-to-moderate learning disability who are not currently on the learning disability register.

**Design and setting:**

An observational study in primary care in West Yorkshire.

**Method:**

Read Code searches were created to identify individuals with a learning disability not on the LD register; they were field tested and further refined before testing in general practice.

**Results:**

Diagnostic codes identified small numbers of individuals who should have been on the LD register. Functional and service use codes often created large numbers of false-positive results. The specific descriptive codes ‘Learning difficulties’ and ‘Referral to learning disability team’ needed follow-up review, and then identified some individuals with LD who were not on the register.

**Conclusion:**

The Read Code search supported practices to populate their registers and was quick to run and review, making it a viable choice to support register revalidation. However, it did not find large numbers of people eligible for the LD register who were previously unidentified by their practice, suggesting that additional complementary methods are required to support practices to validate their registers.

## INTRODUCTION

Individuals with a learning disability (LD) are estimated to constitute 2% of the adult population.^1^ Only around a quarter of affected adults are on the learning disability register at their local general practice.^1^^,^^2^ A learning disability is defined as a:

*‘... significantly reduced ability to understand new or complex information, to learn new skills (impaired intelligence), with a reduced ability to cope independently (impaired social functioning); which started before adulthood, with a lasting effect on development’*.^3^

A learning disability is often classified by IQ scores, with a score of 50–70 defined as mild, 35–50 moderate, 20–35 severe, and <20 as profound.^4^ However, IQ is not a favoured way to define a learning disability because it does not represent the functional abilities people have.^4^ Individuals on the registers tend to be those with more significant/severe intellectual impairment who are known to specialist services.^1^ Less is known about the lives and care needs of adults with milder learning disabilities,^5^ even though those with milder learning disabilities could still benefit from flagging on primary care systems, because they too experience high rates of physical ill health and have a reduced life expectancy.^6^ There is evidence that people with a learning disability are less likely to receive appropriate care for chronic health conditions, including diabetes,^7^^–^9 and tend to have poor self-management abilities,^7^ and worse outcomes than the general population.^10^ Accurate identification and recording of milder learning disability is the first step in correcting this situation.

In the UK, the 2001 government white paper *Valuing People* aimed to reduce health inequalities experienced by this group.^3^ Part of its plan was to introduce a goal that all general practices should hold a register of patients with a learning disability. In 2006, the Disability Rights Commission recommended the introduction of annual health checks for people with learning disabilities in England.^11^ In 2008/2009 a directed enhanced service (DES) introduced health checks for people with a learning disability who were known to social services.^12^ This DES has continued to feature in each contract, and in 2017/2018 there is an increased payment per health check.

Basing health check provision on LD registers that are populated solely on information about people known to social services is inadequate, as it cannot be assumed that the local authority will be aware of all patients with learning disabilities who may qualify for a health check, and those people with a milder disability may not be in receipt of social service support.^13^ There is, therefore, a benefit in practices compiling their own registers for completeness.^12^

Attempts have been made to improve the comprehensiveness of learning disability registers using computerised searches of clinical databases in primary care, but they have focused on diagnostic coding alone.^1^^,^^13^ This paper describes the creation of a new search strategy that goes beyond diagnostic coding to identify patients who have an LD but are not on the practice LD register. The search was originally developed as part of a case-finding exercise for a feasibility randomised controlled trial (RCT), OK Diabetes,^14^ which aimed to identify and recruit adults with a mild-to-moderate learning disability and type 2 diabetes who lived in the community.

**How this fits in**

Learning disability (LD) registers are a key way to identify patients who could benefit from reasonable adjustments to primary care services and/or health checks. LD registers are usually based upon people known to social services, which can mean that those who have a milder disability are not identified on a register but can suffer poor health as a result of their LD. It is not time efficient to explore patient records at length to identify one or two more people with a learning disability. A quick method is required to validate the membership of the LD register. The search created by the authors in this study proved popular as it took only four clicks in the practice system.

## METHOD

### Developing search terms

Searches were devised using standard diagnostic codes (Read Codes) to help practices identify individuals who were potentially eligible but not on a learning disability register. They wanted to create a list of patients whose associated Read Codes indicated they might have a learning disability, or had accessed learning disability services, and who did not have a code that would put them on the LD register. The list of codes was needed as the basis for a search that could be run on any general practice health record system.

The research team held a series of meetings with system specialists and learning disability clinical experts. Developing a candidate list of codes required several meetings, based upon previous studies and clinical experience.^1^^,^^13^^,^^15^^,^^16^ Complexity was added in that even diagnostic codes can have multiple entries in Read Codes. For example, Down’s syndrome can be coded in five ways, two using the term Down’s syndrome and three describing Trisomy 21, which may cause problems for audit and research.^15^

The team decided to focus on four domain categories for the creation of the Read Code search — diagnostic codes, functional codes, service access codes, and descriptive codes.

Diagnosis codes identify conditions often associated with a learning disability but that do not automatically add a person to the LD register — for example, Asperger’s syndrome, Autistic disorder, or Down’s syndrome. Including these codes in a search would ensure the need for a clinician to confirm or discount a learning disability.

Examples of functional codes included ‘difficulty making considered choices’ (XaA3B) and ‘problems with learning’ (ZV400). Service use codes included ‘attendance at special school’ (XM1Zd) and ‘referral to learning disability team’ (XaJmc).

Descriptive codes were those that described the person’s life and status but fell short of a diagnosis and did not describe everyday functioning. They included ‘developmental delay’ (X76B7) and ‘learning difficulties’ (13Z4E).

### Field testing the searches

Working with the data quality team at the Commissioning Support Unit (CSU; this is an external organisation that supports clinical commissioning groups with commissioning evidence and research), the usefulness of certain diagnostic codes was tested by running anonymised searches of data held at CSU level for three cities in West Yorkshire, for people who were not on the LD register but had a diagnostic code indicating a learning disability.

CSU data was searched for one city in West Yorkshire using a wider range of Read Codes.

On the basis of the CSU level searches, the full search was modified, and two general practices then ran the complete search on their systems and made the authors aware of the number of hits each code created.

The search was revised, and two further practices reviewed the cases identified by the searches and fed back the results. When finalised, the search was published into SystmOne (the most prevalent clinical computer system in these areas) under clinical reporting (a full list of codes can be found in Appendix 1). For other systems, it was condensed into a zip file to be e-mailed to practices.

As a final check of the practical utility of the search, practices were invited to use the search to identify participants for referral to the OK Diabetes RCT, and recorded how often referrals resulted from the search.

## RESULTS

### Diagnostic codes at the CSU level

In one city in West Yorkshire (population 500 000), there were 26 people with codes for a diagnosis of Down’s syndrome who were not on the LD register at their practice. Table 1 shows the results when other diagnosis codes were searched for people not on the LD register in three cities in West Yorkshire with a combined population of 1.4 million.

**Table 1.** Diagnostic codes for people not represented on a learning disability register^a^

**Diagnosis code**	**Number not on LD register**
Prader–Willi syndrome	5
Fragile X	31
Autistic disorder	555

a Results from search using three diagnostic codes: numbers identified who were not on the LD register in three cities in West Yorkshire (combined population 1.4 million). LD = learning disability.

### Other Read Codes at the CSU level

Table 2 shows the number of patient records identified by codes that were considered in one city in West Yorkshire (population 500 000), after excluding those individuals on a practice learning disability register.

**Table 2.** Number of patients by code^a^

**Code**	**Number of records (excluding LD register)**
Unable to perform personal care activity	16 256
Declined diabetic retinopathy screening	3038
Learning difficulties	1045
Lives in care home	743
Adult safeguarding concern	710
Impaired cognition	577

a Results from search using five Read Codes: number of patients identified who were not on the LD register in one city in West Yorkshire (population O.5 million). LD = learning disability.

It was agreed the first two terms in the table returned too many results and would damage the specificity of the search. ‘Learning difficulties’ was considered too important a term to remove, despite its high return. ‘Lives in care home’ and ‘Impaired cognition’ were retained for testing purposes. ‘Adult safeguarding concern’ was removed due to concerns that clinicians would feel uncomfortable reviewing and referring this patient.

### Read Codes — practice level

Table 3 shows the results of the search for potential LD when run at Practice 1. The practice found two people who they felt did have a learning disability and were not on their register. Practice 1 has a larger than CCG average list size of approximately 10 000. It resides in the fourth most deprived centile, with a high Quality and Outcomes Framework (QOF) achievement.^17^ The search was conducted by a nurse who leads on learning disabilities in the practice. This practice had updated its LD register 3 years prior to this search, using a list provided by social services. Those individuals who were marked as ‘don’t know’ were flagged with a GP to explore the next time the individual visited the practice.

**Table 3.** Practice-level results of search test

**Number**	**Read Code that identified them**	**In clinical option do they have an LD? yes/no/don’t know, and notes**
1	Receives disability living allowance	No (after some exploration)
2	Asperger’s syndrome	No, just Asperger’s without LD
3	Learning difficulties	No, not clear why this term was on record, possibly dyslexia
4	Asperger’s syndrome	Don’t know
5	**Learning difficulties/cerebral palsy^a^**	**Yes**
6	**Referral to learning disability team^a^**	**Yes**
7	Autistic disorder	No. Just autistic, no LD (clinical decision)
8	Learning difficulties	Don’t know
9	Asperger’s syndrome	No
10	Learning difficulties	Don’t know

a Numbers 5 and 6 in bold show that the practice found two people who they felt did have a learning disability and were not on their register. LD = learning disability.

In the search for potential LD when run at Practice 2, the practice found two people who did have a learning disability and were not on their register, and three who required further review. Practice 2 has a list size of approximately 4000, and it resides in the seventh most deprived centile with a high QOF achievement.^17^ The search was conducted by a GP who leads on learning disabilities in the practice. The search found 14 people who required GP review to exclude — three people with depression, nine people with impaired cognition, one with Asperger’s syndrome, and one attending a voluntary agency. This practice regularly updates its LD register.

### Case finding for RCT

In practice, the simplicity of the search strategy proved popular as it took only four ‘clicks’ to run all of the searches. Members of the research team gave support on the use of the searches if required, but it was rarely asked for.

In all, 65% (*n* = 145) of general practices in the study catchment areas were involved in recruitment to the RCT. Of the 325 participants referred, the most successful methods of identification in primary care was cross-referencing learning and diabetes QOF registers (*n* = 116, 36%). The Read Code searches identified an additional 65 individuals (20%).

## DISCUSSION

### Summary

A population-level search identified relatively small numbers of people who were not on a LD register who had LD diagnosis codes on their records. The findings raised disconcerting questions because even obvious diagnostic codes like Down’s syndrome identified unregistered patients. This suggests the value of a practice-level search strategy where patients can be identified and added to the register.

From the two practices with which the search was developed, the implication is that the Read Code search will identify about two patients per practice who should have been on a learning disability register, at the expense of reviewing about 10 patients who might have other needs for additional support but did not have a learning disability. For a practice with a list size of 5000 (4000 adults), there should be about 80 adults with a learning disability and, typically, about 20–30 will be on the practice LD register. The search, which is easy and quick to use, will add about 10% to the register.

Most useful was the code ‘learning difficulties’, which identified the most patients with a potential learning disability, but is coded to mean a variety of things — mild learning disability or severe dyslexia, for example^1^ — and will therefore include some false positives. ‘Referral to learning disability service’ captured some individuals who were not on the register and therefore proved useful in the study. Diagnostic codes returned few results but would not create many false positives.

Read Code searches alone may be unlikely to identify those individuals with a milder learning disability who continue to experience inequalities in health care and outcomes. Further ways of identifying the hidden majority of adults with milder learning disabilities are required, both to support their inclusion in research and to deliver reasonably adjusted healthcare services to this significantly disadvantaged group.

### Strengths and limitations

The search is quick to run when published to a primary care clinical system such as SystmOne and can result in the identification of new patients. Searches at city population level have shown there is a need for further register revalidation as people are being missed from registers.

The new search does not identify substantial numbers from the hidden majority of unregistered people with a milder learning disability. It is unclear why, but the answer is likely to be because this group do not have a diagnosis and may not self-identify as having a learning disability, and because GPs are reluctant to assign relevant codes even if the patient’s difficulties may be recognised informally when direct contact occurs.

A further limitation of this study is that those clinicians who were willing to review the results were often already interested in learning disabilities, and may have already done significant work on validating their registers. The addition of extra codes not only increased the chances of finding people ‘missed’ by previous searches, but also increases the workload for clinicians who need to review the results.

### Comparison with existing literature

This study developed the work of previous searches to validate LD registers,^1^^,^^12^ including a greater number of codes to increase the sensitivity of the search. The decision to include functional codes responded to previous findings about the variability of LD coding and aimed to capture those individuals without a formal disease diagnosis.^14^ It did not attempt to identify prevalence of LD in the general population. Instead, it focused on finding people not identified by the LD register who may benefit from the reasonable adjustment of services.

### Implications for research and practice

More effective ways of identifying the hidden majority of adults with milder learning disabilities to support their greater inclusion in research need to be found. Current methods are time consuming and require local knowledge on a practice-by-practice basis.^16^ The transfer to SNOMED CT (a structured clinical vocabulary for use in an electronic health record) may support further refinement of searches, but this is currently uncertain. Research is also required to explore how decisions are made about who is included on the LD register.

The DES’s focus on people known to the local authority has meant that the register is often thought of as only for people with a more severe LD.^18^ However, there are people who have a mild or moderate LD who would benefit from, and have a legal right to, reasonable adjustments who are currently not flagged in the system.^19^ This new search is quick, and will identify people currently not offered such adjusted service provision.

## Appendix 1. Potential learning disability (LD) coding

**Table:**

**Name**	**Read Code V3**	**Read Code V2**
[V]Problems with learning	ZV400	N/A
[X]Developmental disorder of scholastic skills, unspecified	Eu81z	N/A
[X]Developmental disorder of scholastic skills, unspecified	Eu81z	Eu81z
Angelman’s syndrome	PKyz5	PKyz5
Asperger syndrome	X00TP	Eu845
Athetoid cerebral palsy or Vogt’s dis: [ophth][neurol]	F1370	F1370
Attendance at special school	XM1Zd	N/A
Attending day centre	XaLLI	N/A
Autistic disorder	XE2v2	N/A
Benign autosomal dominant microcephaly	X77qk	N/A
Chromosomal abnormality	PJ...	PJ...
Classical phenylketonuria	Xa0lA	N/A
Communication assistance from carer requested	XaR79	N/A
Communication skills	XaIyF	N/A
Congenital cerebral palsy	XM1Pu	F23..
Congenital cerebral palsy NOS	F23z.	F23z.
Day care centre	YA265	8GE6.
Declined diabetic retinopathy screening	XaPjM	9m0A.
Delayed reaction time	X765f	N/A
Dependent on others	XaQv2	16ZB3
Developmental delay	X76B7	R034E
Developmental disorder	X00TI	E2Fz
Attended diabetes structured education programme	N/A	9OLB.
Diabetes structured education programme declined	XaNTH	9OLM.
Diabetic patient unsuitable for digital retinal photography	XaKT5	9OLD.
Difficulty analysing information	XaAyZ	N/A
Difficulty comprehending concept of danger	Xa8LF	N/A
Difficulty making considered choices	XaA3B	N/A
Difficulty making decisions	X75x8	N/A
Difficulty making plans	X75x7	N/A
Difficulty performing logical sequencing	XaA2O	N/A
Difficulty processing information	XaAyV	N/A
Difficulty processing information accurately	XaCy8	N/A
Difficulty processing information at normal speed	XaCyB	N/A
Difficulty reasoning	XaA2L	N/A
Difficulty solving problems	X75x5	N/A
Difficulty telling the time	Xa3BA	N/A
Difficulty using arithmetic reasoning	XaA2d	N/A
Difficulty using decision-making strategies	XaA2T	N/A
Difficulty using verbal reasoning	XaA2h	N/A
Difficulty using visuospatial reasoning	XaA2Y	N/A
Diffuse neurofibroma	X78E5	N/A
Domiciliary service need	13V5.	13V5.
Dominated by carer	Ua29k	N/A
Down’s syndrome	XE1MZ	PJ0-98
Down’s syndrome NOS	X78Ek	PJ0z.
Educated at mixed mainstream and special needs school	Ua0SI	N/A
Educated at special needs school	Ua0SG	N/A
Edwards’ syndrome	PJ2..	PJ2..
Edwards’ syndrome NOS	X78Em	N/A
Evidence of lack of understanding	Y1944	N/A
Exc learn disability quality indicators: informed dissent	XaRFM	N/A
Exc learn disability quality indicators: patient unsuitable	XaRFN	9hL1.
Family/carer attended diabetes structured education prog	XaKH1	9hL0.
Family/carer referral to diabetes structured education prog	XaKGz	8Hj1.
Fragile X chromosome	PJyy2	PJyy2
Fragile X syndrome	X78FB	N/A
Global developmental delay	Ua14s	Eu85.
Has contact with multiple support agencies	XaJQr	N/A
Has difficulty with speech	1B93.	1B93.
Help by relatives	13WJ.	13WJ.
Home help attends	13G61	13G61
Hydromicrocephaly	P210.	P210.
Impaired cognition	Ua189	N/A
Infantile autism NOS	E140z	E140z
Informed dissent for diabetes national audit	XaJrE	9M10.
Intellectual functioning disability	Ub0ih	N/A
Klinefelter syndr: [male, more than 2 X chrom][XXXY][XXXXY]	PJ71.	N/A
Klinefelter’s syndrome	PJ7..	N/A
Klinefelter’s syndrome NOS	PJ7z.	PJ7z.
Klinefelter’s syndrome XXXXY	XM1MK	N/A
Klinefelter’s syndrome XXXY	XM1MJ	N/A
Klinefelter’s syndrome XXYY	PJ73.	PJ73.
Klinefelter’s syndrome XY/XXY mosaic	PJ74.	PJ74.
Klinefelter’s syndrome — male with more than two X chromosomes	XE1Mg	PJ71.
Learning difficulties	13Z4E	13Z4E
Learning disabilities administration status	XaJW7	N/A
Learning disabilities annual health assessment	XaL3Q	9HB5.
Learning disabilities health action plan completed	XaJsd	9HB4
Learning disabilities health action plan declined	XaJW9	9HB0.
Learning disabilities health action plan offered	XaJW8	9HB1.
Learning disabilities health action plan reviewed	XaJWA	9HB2.
Learning disabilities health assessment	XaJmb	9HB3.
Lives in a welfare home	13F71	13F71
Lives in care home	XaMFG	13FX.
Lives in staffed home	Ua0Lj	N/A
Lives in supported home	Ua0Le	N/A
Memory: present time not known	N/A	3A20.
Mental handicap problem	6664	6664
Micrencephaly	P211.	P211.
Microcephalus NOS	P21z.	P21z.
Microcephaly	P21..	P21..
Mild cognitive impairment	N/A	28E0
Moderate cognitive impairment	N/A	28E1
Multiple system congenital anomalies NEC	XE1Ml	PKy0.
Needs help with cooking	XaIwv	39G0.
Needs help with housework	XaIwu	39G..
Neu–Laxova syndrome	Xa0ZQ	N/A
Neurofibromatosis	Xa99T	N/A
Neurofibromatosis type 1	B927.	B927.
Neurofibromatosis type 1	B927.	N/A
Neurofibromatosis type 2	X78E2	N/A
Neurofibromatosis type 3	X78E3	N/A
Noonan’s syndrome	PKy80	PKy80
Not involved in dealing with own monies	Ua29y	N/A
Other accommodation with care and support not specialist mental health	Y0adf	N/A
Other skill difficulties	Y2617	N/A
Parent provides full-time care	XaQ8Y	N/A
Parent provides part-time care	XaQQV	N/A
Partial trisomy 18 in Edwards’ syndrome	X78En	N/A
Partial trisomy 21 in Down’s syndrome	X78El	N/A
Phenylketonuria	C301.	C301.
Prader–Willi syndrome	PKy93	PKy93
Preferred method of communication: Makaton	XaR76	N/A
Preferred place of care — learning disability unit	XaR4m	N/A
Primary microcephaly	XM00P	N/A
Problem related to life management difficultly, unspecified	N/A	ZVu5C
Receives help from friend	Ua0VF	N/A
Care from friends	N/A	8GEB.
Receives help from lay carer	Ua0VD	N/A
Receives help from neighbour	Ua0VH	N/A
Receives help from relative	Ua0VG	N/A
Care from relatives	N/A	8GEA
Receives help from voluntary agency	Ua0VE	N/A
Referral to learning disability team	XaJmc	8HHP.
Requires communication partner	XaJHX	N/A
Rett syndrome	Eu842	Eu842
Secondary microcephaly	X77ql	N/A
Segmental neurofibromatosis	X78E4	N/A
Severe cognitive impairment	N/A	28E2
Slow flow of thought	X75xy	N/A
Slow learner	Ua187	N/A
Problems with learning	N/A	ZV400
Special educational needs	Ub0gW	N/A
Special educational plan in place	Y4850	N/A
Speech limited	1B441	1B441
Sturge–Weber syndrome	PK61.	PK61.
Supported accommodation	Y0ad2	N/A
Supported group home	Y0ad4	N/A
Supported lodgings	Y0ad3	N/A
Suspected autism	XaIuT	N/A
Trisomy 21 — meiotic nondisjunction	PJ00.	PJ00.
Trisomy 21 — mitotic nondisjunction mosaicism	PJ01.	PJ01.
Trisomy 21, translocation	N/A	PJ02.
Tuberous sclerosis	PK5..	PK5..
Unable to analyse information	XaAyY	N/A
Unable to comprehend concept of danger	Xa8LE	1BV1.
Unable to express self	Xa3Y1	N/A
Unable to make considered choices	XaA3A	28Q1.
Unable to manage medication	Xa2yD	N/A
Unable to perform logical sequencing	XaA2P	N/A
Unable to perform shopping activities	Xa7h1	N/A
Unable to plan	Xa3bT	N/A
Unable to process information	XaAyU	N/A
Unable to process information accurately	XaCy7	N/A
Unable to process information at normal speed	XaCyA	N/A
Unable to read	XaBmf	N/A
Unable to reason	XaA2K	N/A
Unable to tell the time	Xa3BD	N/A
Unable to think clearly	X75yC	N/A
Unable to use arithmetic reasoning	XaA2c	N/A
Unable to use decision-making strategies	XaA2S	N/A
Unable to use medication	N/A	8BIj.
Unable to use verbal reasoning	XaA2g	N/A
Unable to use visuospatial reasoning	XaA2X	N/A
Unable to write	XaAzP	N/A
Voluntary worker attends	XE0p5	N/A
Williams syndrome	PKy4.	PKy4.
XXY Klinefelter’s syndrome	PJ70.	PJ70.
XXY Klinefelter’s syndrome	PJy3.	PJy3.

Exc = excluding. LD = learning disability. NEC = not elsewhere classified. NOS = Not otherwise specified.
